# Supplementary material for: Electrophysiological network alterations in adults with copy number variants associated with high neurodevelopmental risk
Source: Transl Psychiatry. 2020 Sep 21;10:324. doi: 10.1038/s41398-020-00998-w (PMC7506525; doi:10.1038/s41398-020-00998-w)
Supplement: Supplementary file 1 — Supplementary Information [file 41398_2020_998_MOESM1_ESM.pdf]

Supplementary Information

	ND-CNV vs Controls (whole cohort)							22q11.2del vs other ND-CNV
	Eccentricity	*Degree*	*Betweenness centrality*	*Global efficiency*	*Local efficiency*	*Clustering coefficient*	*Pooled node features*	*Pooled node features*
**Accuracy (%)**	68.64	63	58.84	63.23	63.46	60.43	71.38	72.88
**SD**	2.77	3.77	3.76	3.75	3.82	3.54	2.98	1.58
**P-value**	0.0002	0.0008	0.0144	0.0008	0.0008	0.0086	0.0002	0.0036
**Sensitivity**	0.59	0.59	0.55	0.6	0.6	0.56	0.68	0.68
**Specificity**	0.79	0.67	0.63	0.66	0.67	0.65	0.74	0.76
	**ND-CNV vs Controls (excluding 22q11.2del and matched controls)**							**Train on 22q11.2del, test on other ND-CNV**
**Accuracy (%)**	65.02	61.02	51.93	56.59	57.52	54.73	62.84	55.36
**SD**	4.28	4.78	5.01	5.08	4.49	5.3	4.23	-
**P-value**	0.0022	0.0166	0.3601	0.1086	0.0618	0.1668	0.0128	0.225
**Sensitivity**	0.56	0.56	0.5	0.55	0.55	0.52	0.58	0.21
**Specificity**	0.74	0.66	0.54	0.58	0.61	0.57	0.67	0.89
	**ND-CNV vs Controls (22q11.2del vs matched controls only)**							**Train on other ND-CNV, test on 22q11.2del**
**Accuracy (%)**	76.08	64.49	66.07	73.95	84.91	89.02	85.17	75
**SD**	4.65	5.82	7.26	5.48	3.95	4	4.17	-
**P-value**	0.0006	0.0332	0.0112	0.0012	0.0002	0.0002	0.0002	0.006
**Sensitivity**	0.6	0.54	0.61	0.63	0.77	0.84	0.78	0.57
**Specificity**	0.92	0.75	0.72	0.85	0.93	0.94	0.92	0.93

Supplementary Table 1. Network-based classification results. Note that cross-decoding across ND-CNV subgroups achieves high specificity but not sensitivity (middle-right and lower-right cells). This suggests that classifier performance is driven mainly by network features in the control group.

For classification between participants with 22q11.2 deletions and other ND-CNVs (top right cell), 100 subsamples of 14 participants were drawn from the 28 carriers of other ND-CNVs, gender-matched to the 22q11.2 deletion group. The subgroups were similar in age, ensuring balanced classes in terms of sample size, gender, and age. Predicted labels were then averaged across cohort resamplings (mean 50±18.8 repetitions per participant).

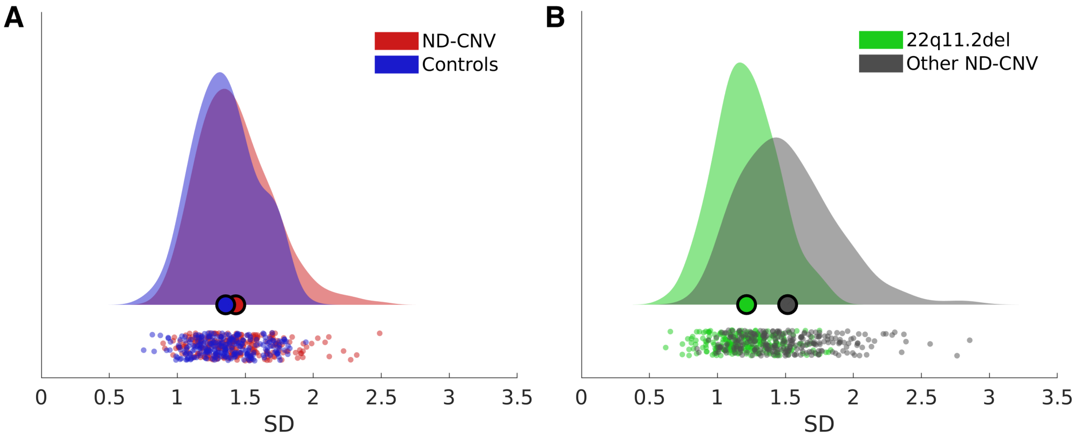

Supplementary Figure 1. Variability in connection z-scores across participant groups. The distribution of standard deviations (SD) across valid connections in the combined frequency maps are shown for: **A.** the ND-CNV and control groups; **B.** the two ND-CNV subgroups. The mixed ND-CNV group displays larger variability. Plots were generated using the RainCloudPlots toolbox[1].

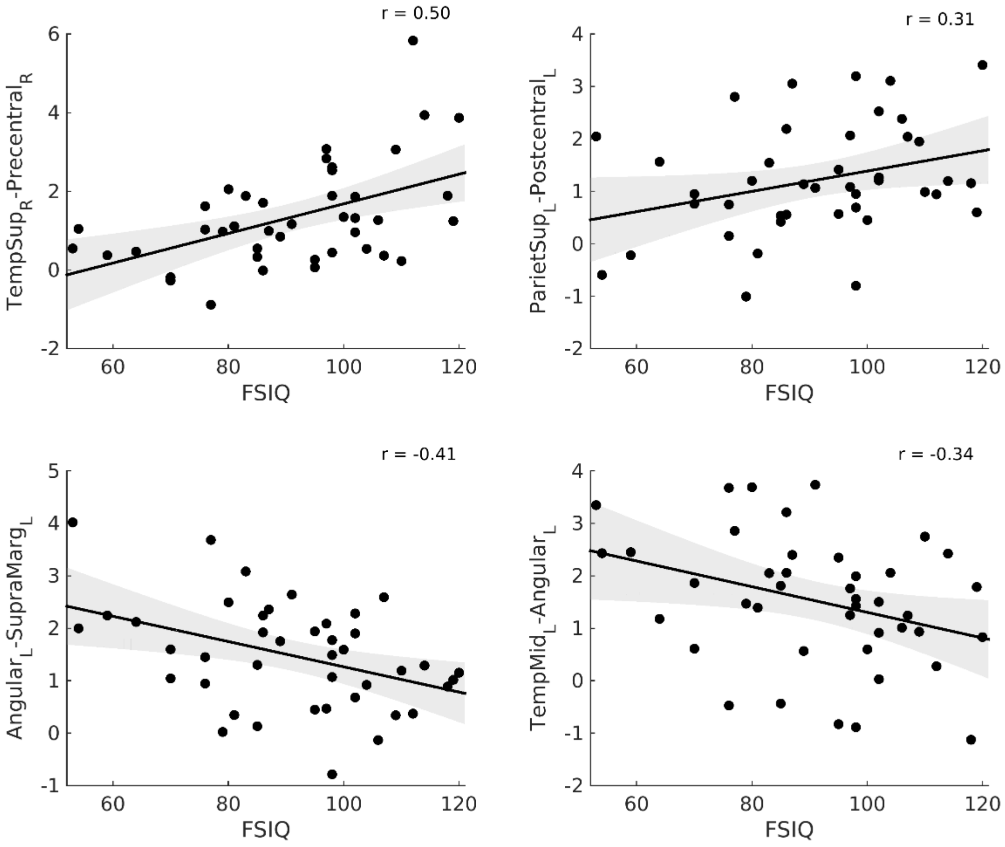

Supplementary Figure 2. Impact of FSIQ scores on connectivity results. The relationship between connectivity strength (combined-frequency maps) and FSIQ is shown for the only four connections where a significant correlation was found (P<0.05). Note that despite the presence of 6 participants with intellectual disability, the median IQ of the ND-CNV group was within the average range (VIQ:90; PIQ:100; FSIQ: 95).

[1]Allen M, Poggiali D, Whitaker K, Marshall TR, Kievit RA (2019) Raincloud plots: A multi-platform tool for robust data visualization [version 1; peer review: 2 approved]. Wellcome Open Res 4.

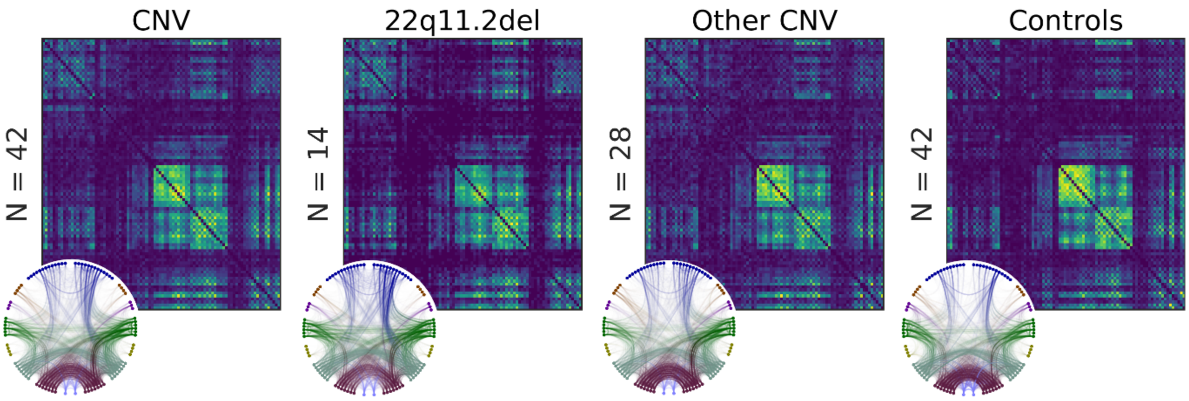

Supplementary Figure 3. Average connectivity matrices for each group of subjects after individual rank thresholding (stronger edges in yellow). Individual thresholded matrices were used as the basis for graph construction.

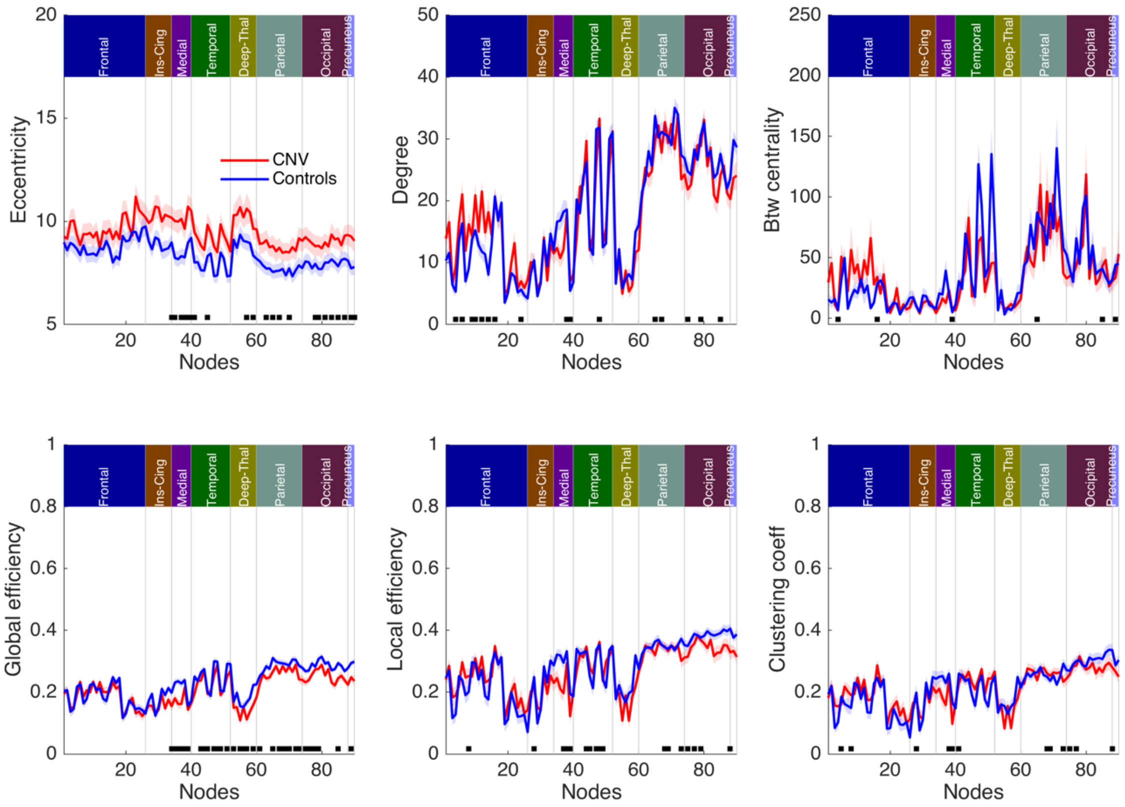

Supplementary Figure 4. Node features in the ND-CNV cohort compared to controls. Nodes are organized according to region as in the circular plots (see Supplementary Figure 1). Error bars are ±SEM. Note that better-connected regions (e.g. higher node degree values) generally correspond to valid connections in the group analysis (occipital, parietal, temporal). Black markers show nodes with a significant between-group difference (unpaired t-test, uncorrected α = 0.05).

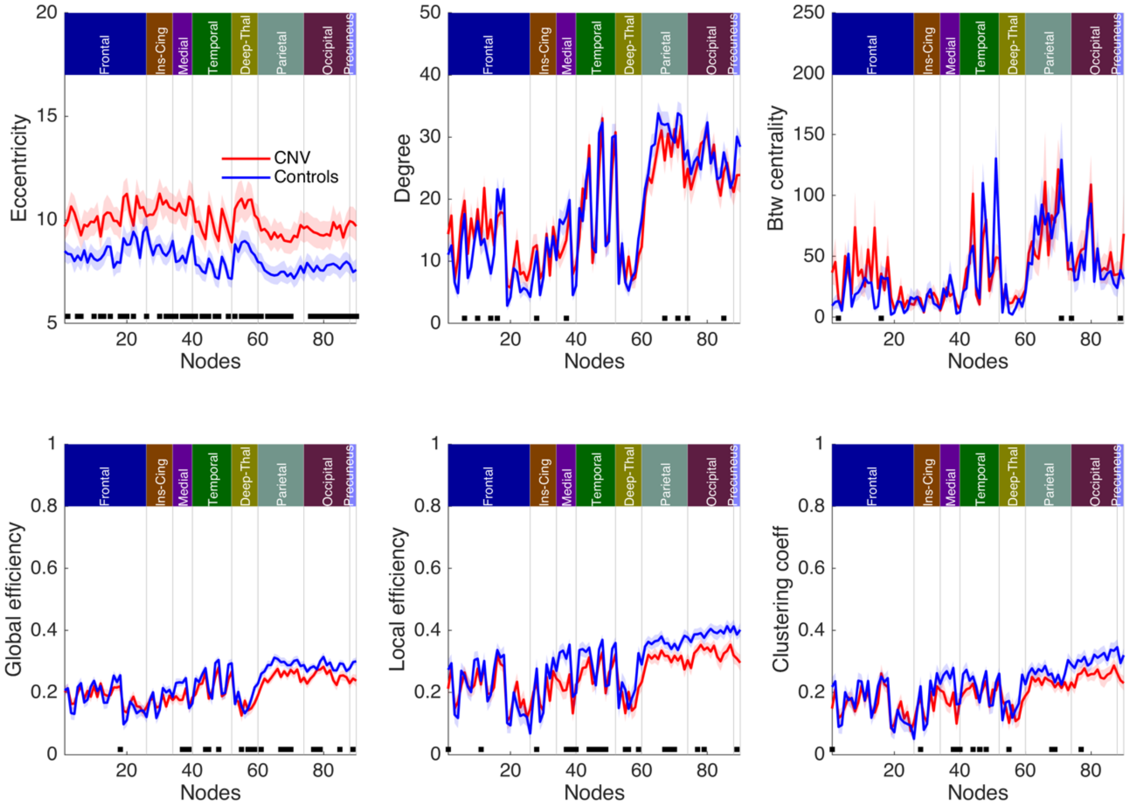

Supplementary Figure 5. Node features in the ND-CNV cohort after excluding participants with 22q11.2 deletions, compared to their matched controls.

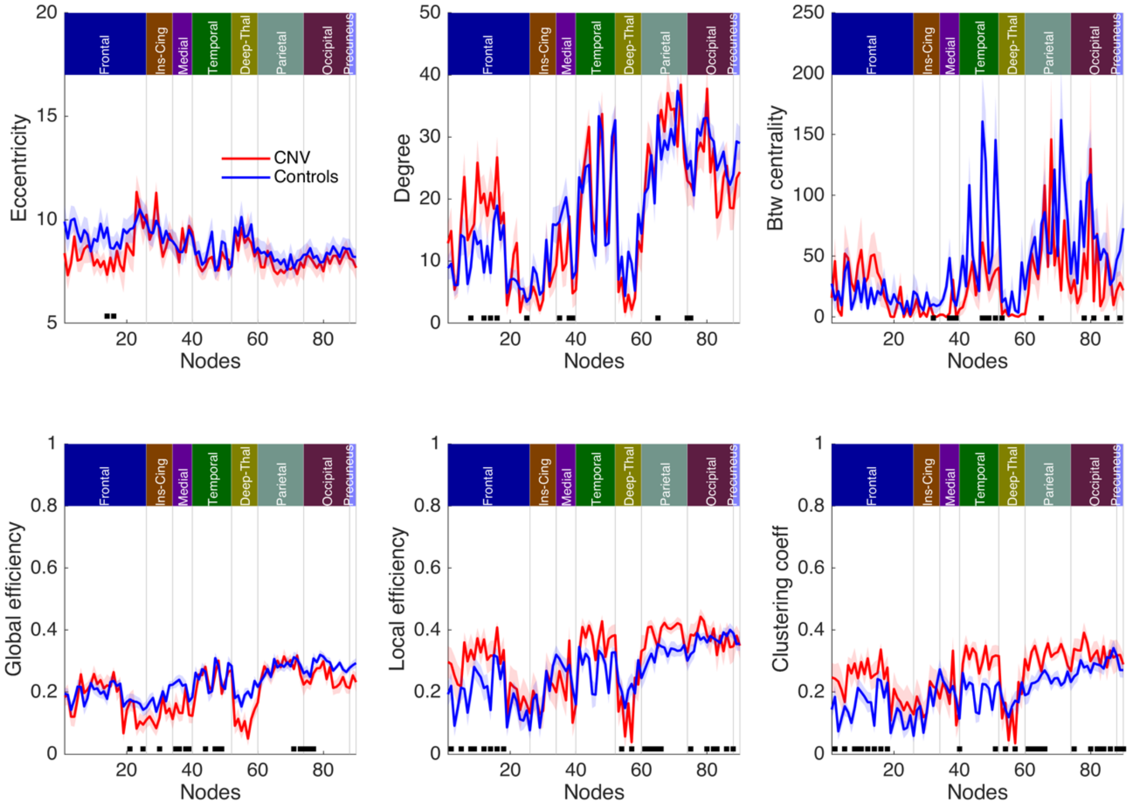

Supplementary Figure 6. Node features in the 22q11.2 deletion group compared to their matched controls.

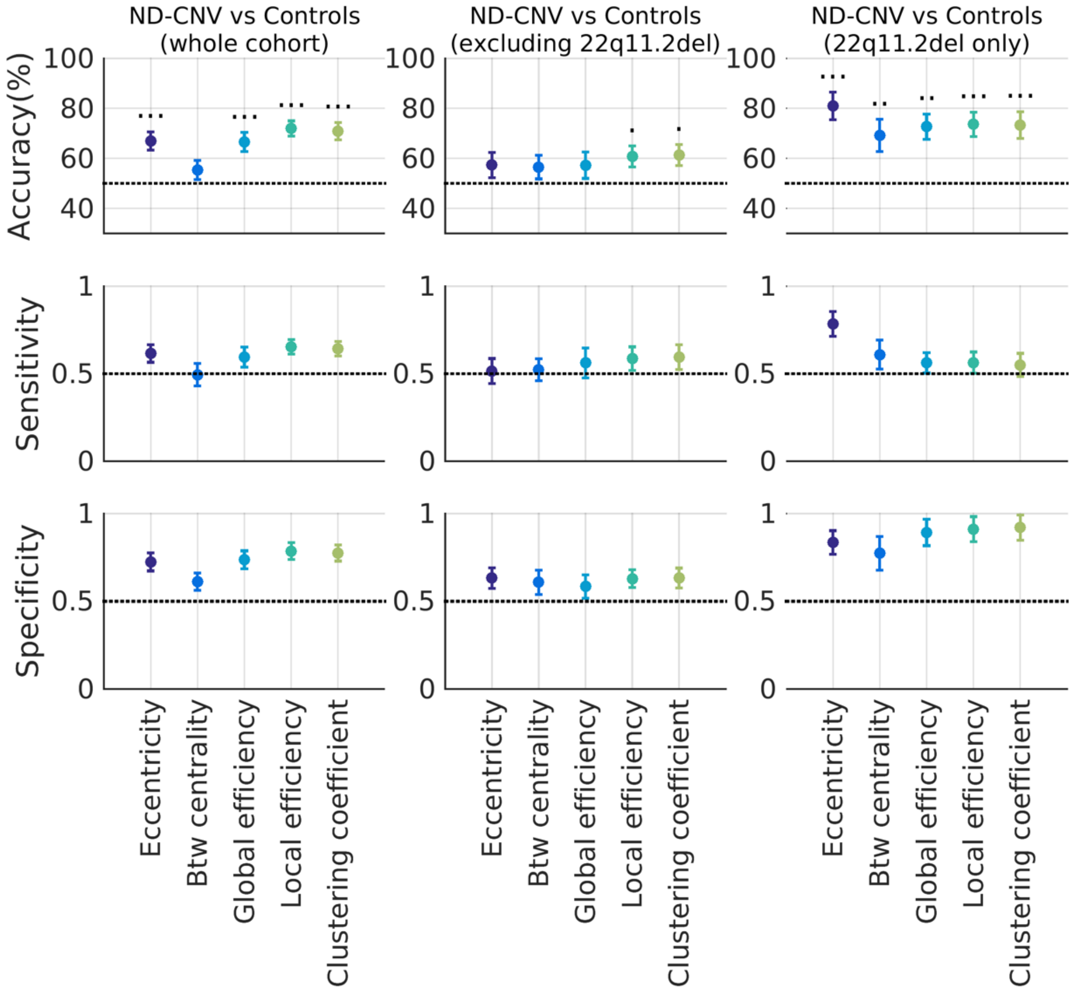

Supplementary Figure 7. Calculating the weighted graph theory metrics (all metrics except node degree) based on unthresholded connectivity maps leads to a largely similar pattern of decoding results.
